# Supplementary material for: Bio-Inspired Hierarchical Micro-/Nanostructures for Anti-Icing Solely Fabricated by Metal-Assisted Chemical Etching
Source: Micromachines (Basel). 2022 Jul 7;13(7):1077. doi: 10.3390/mi13071077 (PMC9317431; doi:10.3390/mi13071077)
Supplement: Supplementary file 1 [file micromachines-13-01077-s001.zip › micromachines-1800933-supplementary.pdf]

micromachines

Supplementary Material

Bio-Inspired Hierarchical Micro-/Nanostructures for Anti-Icing Solely Fabricated by Metal-Assisted Chemical Etching

Lansheng Zhang [1], Xiaoyang Chu [1], Feng Tian [1,2], Yang Xu [2] and Huan Hu [1,3,*]

[1] ZJU-UIUC Institute, International Campus, Zhejiang University, Haining 314400, China; lansheng.20@intl.zju.edu.cn (L.Z.); xiaoyang.20@intl.zju.edu.cn (X.C.); tianfeng@zju.edu.cn (F.T.)
[2] School of Micro-Nano Electronics, Zhejiang University, Hangzhou 310000, China; yangxu-isee@zju.edu.cn
[3] State Key Laboratory of Fluid Power and Mechatronic Systems, Zhejiang University, Hangzhou 310000, China
* Correspondence: huanhu@intl.zju.edu.cn

Citation: Zhang, L.; Chu, X.; Tian, F.; Xu, Y.; Hu, H. Bio-Inspired Hierarchical Micro-/Nanostructures for Anti-Icing Solely Fabricated by Metal-Assisted Chemical Etching. *Micromachines* **2022**, *13*, 1077. https://doi.org/10.3390/mi13071077

Academic Editor: Lucia Romano

Received: 19 June 2022
Accepted: 5 July 2022
Published: 7 July 2022

Publisher's Note: MDPI stays neutral with regard to jurisdictional claims in published maps and institutional affiliations.

Copyright: © 2022 by the authors. Submitted for possible open access publication under the terms and conditions of the Creative Commons Attribution (CC BY) license (https://creativecommons.org/licenses/by/4.0/).

Table S1. Different methods to form hierarchical micro/nanostructured surfaces.

Literature Reports	Microstructure Fabrication	Nanostructure Fabrication
Nanosecond pulsed laser irradiation [1]	Nanosecond pulsed laser irradiation	Nanosecond pulsed laser irradiation
Femtosecond [2]	Femtosecond	Hydrothermal treatments
Nanoimprinting [3]	Nanoimprinting with a microtemplate	Nanoimprinting
Hot-embossing [4]	Hot-embossing	Hot-embossing
Laser scanning probe lithography [5]	Scanning probe lithography and wet chemical etching	Scanning probe lithography and wet chemical etching
Glancing angle deposition [6]	Deep reactive ion etching	Glancing angle deposition
Metal-assisted chemical etching (this work)	Metal-assisted chemical etching	Metal-assisted chemical etching

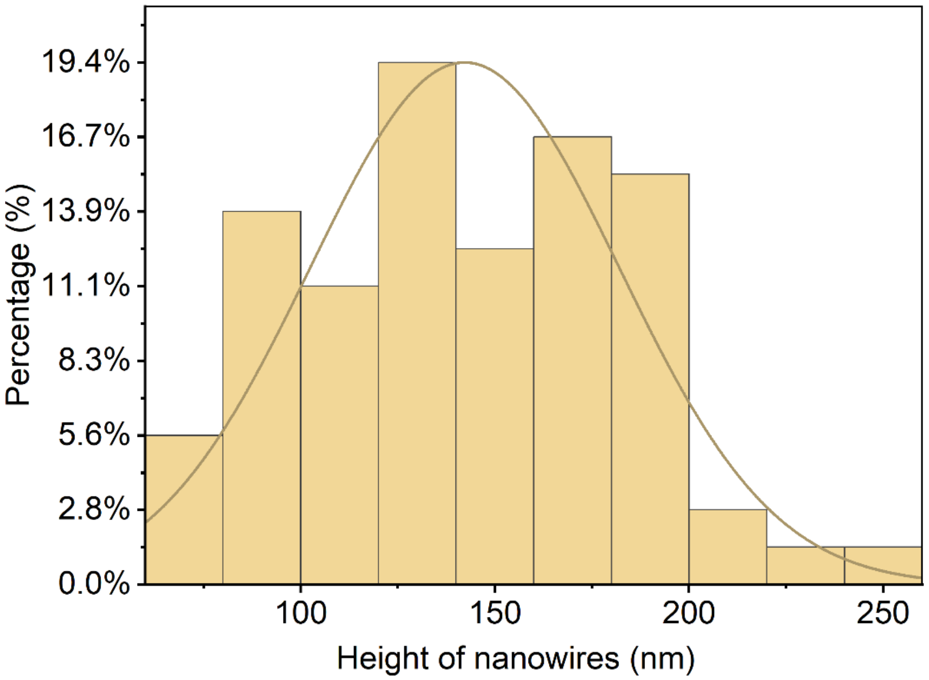

Figure S1. The statistical data of the nanowire heights.

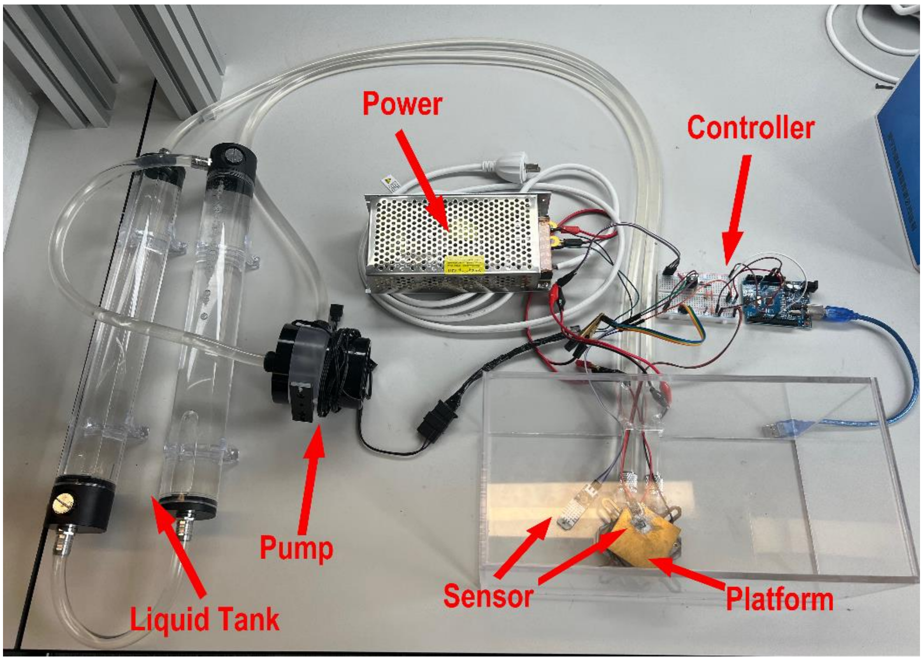

Figure S2. The anti-icing setup for measuring the ice delay time (IDT).

References

1. Wang, C.; Huang, H.; Qian, Y.; Zhang, Z.; Yan, J. One-step fabrication of regular hierarchical micro/nano-structures on glassy carbon by nanosecond pulsed laser irradiation. *Journal of Manufacturing Processes* **2021**, *62*, 108-118, doi:10.1016/j.jmapro.2020.12.043.
2. Lu, J.; Yang, J.; Singh, S.C.; Zhan, Z.; Yu, Z.; Xin, W.; Huang, T.; Guo, C. Hierarchical micro/nanostructured TiO2/Ag substrates based on femtosecond laser structuring: A facile route for enhanced SERS performance and location predictability. *Applied Surface Science* **2019**, *478*, 737-743, doi:10.1016/j.apsusc.2019.01.257.
3. Wang, C.; Shao, J.; Lai, D.; Tian, H.; Li, X. Suspended-Template Electric-Assisted Nanoimprinting for Hierarchical Micro-Nanostructures on a Fragile Substrate. *ACS Nano* **2019**, *13*, 10333-10342, doi:10.1021/acsnano.9b04031.
4. Lim, C.H.; Han, S.Y.; Eo, J.D.; Kim, K.; Kim, W.B. Superhydrophobic hierarchical structures produced through novel low-cost stamp fabrication and hot embossing of thermoplastic film. *Journal of Mechanical Science and Technology* **2015**, *29*, 5107-5111, doi:10.1007/s12206-015-1108-8.
5. Choi, I.; Kim, Y.; Yi, J. Fabrication of hierarchical micro/nanostructures via scanning probe lithography and wet chemical etching. *Ultramicroscopy* **2008**, *108*, 1205-1209, doi:10.1016/j.ultramic.2008.04.076.
6. Zhang, L.; Uzoma, P.C.; Xiaoyang, C.; Penkov, O.V.; Hu, H. Bio-Inspired Hierarchical Micro/Nanostructured Surfaces for Superhydrophobic and Anti-Ice Applications. *Front Bioeng Biotechnol* **2022**, *10*, 872268, doi:10.3389/fbioe.2022.872268.
